# The Screening of *Rubella Virus*, *Cytomegalovirus*, *Hepatitis B Virus*, and *Toxoplasma gondii* Antibodies in Prepregnancy and Reproductive-Age Women in Tabriz, Iran

**DOI:** 10.1155/2022/4490728

**Published:** 2022-01-10

**Authors:** Edris Nabizadeh, Anahita Ghotaslou, Behnaz Salahi, Reza Ghotaslou

**Affiliations:** ^1^Infectious and Tropical Diseases Research Center, Tabriz University of Medical Sciences, Tabriz, Iran; ^2^Student Research Center, Medipol University, Ankara, Turkey; ^3^Razi Hospital, Tabriz University of Medical Sciences, Tabriz, Iran

## Abstract

**Objectives:**

The organisms of *Toxoplasma gondii*, *Rubella virus*, *Cytomegalovirus*, and *Herpes simplex virus* as an acronym of TORCH are major pathogens in prepregnancy and reproductive-age women. These microorganisms are considered a serious problem and cause 2-3% of all birth defects in the fetus. Our study was aimed at screening the seroprevalence of TORCH antibodies among prepregnancy and reproductive-age women in Tabriz, Iran. *Design and Setting*. This study was carried out in 2726 prepregnancy and reproductive-age women, who were referred to the laboratory for prenatal TORCH screening. To detect the presence of IgG, IgM antibodies and Hepatitis B surface antigen against these microorganisms were carried out using a chemiluminescence immunoassay analyzer (CLIA).

**Results:**

In the current study, the rates of anti-*Toxoplasma gondii* IgG, anti-*Rubella virus* IgG, and anti-*Cytomegalovirus* IgG were found in 722 cases (26.5%), 2579 cases (94.6%0), and 2718 cases (99.7%), respectively. Moreover, the rates of anti-*Toxoplasma gondii* IgM, anti-*Rubella virus* IgM, and anti-*Cytomegalovirus* IgM were discovered in 10 cases (0.4%), 13 cases (0.5%), and 16 cases (0.6%), respectively. The Hepatitis B surface antigen was found in 32 cases (1.2%). The dissemination of positive TORCH in various ages was different (*P* < 0.05).

**Conclusions:**

In our study, the seroprevalence of acute TORCH infections was relatively low. Due to the probability of vertical transmission to the fetus during pregnancy and the unpleasant complication of these pathogens, it is essential to be screened for detection of specific IgG and IgM antibodies in reproductive ages.

## 1. Introduction

The infections of Toxoplasma gondii (TOX), Rubella virus (RUV), Cytomegalovirus (CMV), and Herpes simplex virus (HSV), as an acronym of TORCH, are major pathogens with serious problems in prepregnancy and reproductive-age women. They are considered to cause 2-3% of all birth defects in the fetus. Moreover, the letter “o” of TORCH as an abbreviation that refers to others can include some pathogen agents such as Arbovirus B19, Hepatitis B virus (HBV), Varicella-zoster virus, human immunodeficiency virus, and Zika virus [1, 2]. The seroprevalence of these infections is different in various geographical areas [3]. Disease caused by TOX is generally with no sign and with mild infection in healthy individuals, and it may lead to serious problems in the fetus, which result in stillbirth, blindness, and mental retardation [4]. Rubella appears as a simple disease with mild symptoms and usually no specific complication. Despite these findings, whenever it occurs in a prepregnancy and reproductive-age woman, it can cause death and abortion [5]. CMV is one of the most common viral agents causing infection in utero, and its prevalence has been estimated at 0.3-2.4% of all successful deliveries. Infection due to CMV can be associated with severe complications that can eventually lead to growth retardation, jaundice, hepatosplenomegaly, and intracerebral damage [6, 7]. Viral hepatitis caused by some viruses such as *Hepatitis B virus* is a common form of the disease worldwide. Chronic hepatitis patients can transmit the virus for years without any symptoms. Prepregnancy and reproductive-age mothers are believed to be the most important risk factor for transmitting the virus to the fetus. Nowadays, to screen HBV infections in prepregnancy and reproductive-age women, the HBsAg test is recommended [8]. It is a normal initial detection of the TORCH pathogen which is important for treatment and prevention strategies for adverse fetal outcomes [9]. The characteristic of these pathogens is that they are asymptomatic at the beginning of the infection, and they cannot be diagnosed based on their clinical features. The tracing of the IgM antibody against TORCH pathogens is the best aspect of the recognition of these infections [10]. This study was aimed at screening the seroprevalence of TORCH infections among prepregnancy and reproductive-age women in Tabriz.

## 2. Materials and Methods

### 2.1. The Patients

This study was carried out on 2726 prepregnancy and reproductive-age women, who were referred to the laboratory for prenatal screening in Tabriz, Iran, from 2018 to 2020. Awareness consent was received from all women who were referred to screening for TORCH. Blood samples were collected from each person and were centrifuged at 1200 × g for 15 min to separate the serum from the blood.

### 2.2. The Screening

To detect the presence of anti-CMV, TOX, RUV IgG, and IgM antibodies and also HBsAg, the serologic testing was carried out using a chemiluminescent immunoassay (Liaison® XL, Italy). According to the manufacturer's instructions (Liaison Company, Italy), the analysis of the test results was carried out based on the cut-off activity index. Additionally, the quality control process of the device and kits was performed according to the relevant standards.

### 2.3. Statistical Analysis

Statistical analysis was carried out by SPSS 26 (SPSS Inc., Chicago, Illinois, US).

## 3. Results

To determine the seroprevalence of TORCH, this study was conducted in 2726 cases of reproductive-aged women. The mean age of women was 30 ± 7 years old (range 14-45). As shown in Table 1, all women based on age were divided into three categories, and the seroprevalence of TORCH was determined in each category. The distribution of positive TORCH in various ages was different (*P* < 0.05). The rate of anti-*TOX* IgG antibody, anti-*Rubella* IgG, and anti-CMV IgG were found in 722 cases (26.5%), 2579 cases (94.6%), and 2718 cases (99.7%), respectively. Moreover, the rate of anti-*TOX* IgM antibody, anti-*Rubella* IgM, and anti-*CMV* IgM was discovered in 10 cases (0.4%), 13 cases (0.5%), and 16 cases (0.6%), respectively. The seroprevalence of HBsAg *Hepatitis B* virus (HBsAg positive) as other pathogen-related to TORCH was in 32 (1.2%) cases. The seroprevalence of tested TORCH pathogens among 2726 women is shown in Table 2.

## 4. Discussion

Intrauterine infections related to TORCH pathogens can lead to serious fetal complications, despite most of these organisms not being associated with a serious injury in the mothers and appearing often as a mild conflict. Conventional screening via serological assays is effective in the detection of TORCH infections, before pregnancy, prepregnancy, and reproductive-age women. In this investigation, we had analyzed the seroprevalence of TORCH agents (TOX, RUV, CMV, and *Hepatitis B* virus) among prepregnancy and reproductive-age women in Tabriz, Iran. This issue is helpful for the understanding of the diverse TORCH agents spreading in this area. The strength of our research may be a high sample size over a short study period of two years, and it was conducted for the first time in this geographical area. The weakness of our work may be the lack of full patient's follow-up, which will be pursued in the future and lack of definitive confirmation of positive cases by amniocentesis sampling. Due to various factors, the exact spread of TORCH infections is still unknown in most parts of the world [2]. In the present study, acute infections due to TOX, CMV, and RUV were 0.4%, 0.5%, and 0.6%, respectively. *Toxoplasma gondii* is an intracellular pathogen that is transmitted in a variety of ways, including consuming uncooked meat, contact with some animals especially cats, and contaminated foodstuffs or water. Disease caused by this agent can lead to serious problems in the fetus [11, 12]. In the current study, the positive cases were detected for anti-TOX IgG (26.5%) and IgM (0.4%). These findings were different from the average found in previous studies in Iran that reported 35-41%. The reason for this discrepancy may be due to low sample size in the previous study [13]. In addition, the seroprevalence of TOX infections is different in various countries, for example, UK 9.1-7.7% [14], India 28% [12], and Canada 59.8% [15]. This difference in the frequency is likely due to the nutritional habits (consuming well-cooked food and consumption of frozen meat), socioeconomic state, geographical difference, and improved level of health in meat production [16]. *Rubella* appears as a simple viral disease with mild symptoms or is even asymptomatic in neonates and not common in adults [17]. On the other hand, the prevalence of RUV infections in developed countries has been few due to vaccination programs. Previous studies have reported the seroprevalence of RUV in prepregnancy and reproductive-age women in several countries ranging from 83.4 to 97.9%, for example, India (83.4%) [18], southern Italy (85.8%) [19], Turkey (96.3%) [20], and Nigeria (97.9%) [21]. The results of our study revealed that 89.4% of the women had anti-RUV IgG antibodies and also 0.5% had Rubella IgM. These results are almost in agreement with other studies' findings in some parts of Iran, which suggest the success of the vaccination program [22]. RUV disease usually occurs in cases that come from countries without a vaccination program against this virus or people who refuse to be vaccinated.

CMV is one of the most common viral agents classified in the herpes family. This pathogen can be associated with severe complications that can eventually lead to growth retardation, jaundice, hepatosplenomegaly, and intracerebral damage. The virus is also found globally in developed and underdeveloped countries, especially in areas with a high economic crisis. Its seroprevalence is estimated between 45% and 100% and 0.2-2.2% morbidity [23]. The current study indicated that the anti-CMV IgG antibody in 99.7% cases, as well as 0.6% of women, had a positive IgM antibody. In a study from Iran carried out in 2015, the prevalence of anti-CMV-IgG and IgM antibodies during the first trimester of pregnancy was reported as 98.8% and 5%, respectively [22]. Besides, in the previous investigations, the seropositive rates of CMV IgG in prepregnancy and reproductive-age women in the various counties were revealed to be different, for example, 84.5-95% in Turkey, 84% in Spain, 56.8 in Australia, and 39-94.7% in the USA [24]. One of the important public health issues worldwide is HBV infection. According to some reports, an estimated 325 million persons in the world were infected with this virus. HBV is a contagious infection that can affect neonates via a vertical transmission [25]. The universal seroprevalence of *HBV* infection is varied and classified based on the seroprevalence in four categories including ≥8% which indicates a high level of infection, 5-7.9% high-intermediate, 2-4.9% low-intermediate, and <2% low [26]. Studies on the prevalence of *Hepatitis B* in prepregnancy and reproductive-age women in some countries are as follows: USA 0.38%, in comparison with foreign women who were born in Asia 2-8.7% [27] and Ghana 7.7% [28]. In the current study, the seroprevalence of HBsAg was 1.2%. Our finding is equal to the result (1.21%) of the study carried out by Shoghli et al., which was performed in seven provinces of Iran [29]. Also, the seropositivity rate of HBsAg in our study was higher than in different cities of Iran, including Babol (0.18%), Dehloran (0.59), and Amol (0.62%) [29, 30]. Moreover, the finding of our study almost was comparable with a survey conducted by Tanrıverdi et al., in Turkish prepregnancy and reproductive-age women, in our neighbor country (1.2%) [31], which probably refers to the hypothesis of ethnic diversity and genetics as a risk factor [32]. But, further studies are needed to prove this hypothesis. Considering TORCH as major pathogens in prepregnancy and reproductive-age women, which is the cause of 2-3% of all birth defects in the fetus and serious problems in the fetus, the screening of TORCH is important. Since these diseases are asymptomatic in a prepregnancy and reproductive-age woman and can be transmitted to the fetus, its normal, initial recognition is vital for the prevention and treatment of diseases due to TORCH. On the other hand, molecular tests are not fully available to them; therefore, we rely on serology testing. On the other hand, the prevalence of TORCH infection varies in different regions, and the prevalence may be high in other parts of the country, so conducting such studies for epidemiological purposes is important. In our study, the seroprevalence of acute TORCH infections was relatively low. However, due to the vertical transmission to the fetus during pregnancy and the unpleasant complication of these pathogens, it is essential to take all necessary precautions, and screening in women must be done at reproductive ages.

## 5. Conclusion

In our study, the seroprevalence of acute TORCH infections was relatively low. Due to the probability of vertical transmission to the fetus during pregnancy and the unpleasant complication of these pathogens, it is essential to be screened by detection of specific IgG and IgM antibodies in reproductive ages.

## Figures and Tables

**Table 1:** The seroprevalence of TORCH IgG and IgM among 2726 women who categorized based on age.

Pathogen	TOX				RUV				CMV				HBV	
Age group	IgG		IgM		IgG		IgM		IgG		IgM		HBsAg	
14-24 y (653 cases)	P	N	P	N	P	N	P	N	P	N	P	N	P	N
	109	544	0	653	545	108	2	651	648	5	6	647	1	652
25-35 y (1361)	353	1008	8	1353	1339	22	11	1350	1358	3	10	1351	16	1345
36-45 y (712)	260	452	2	712	695	17	0	712	712	0	0	712	15	697

**Table 2:** The seroprevalence of tested TORCH pathogens among 2726 women.

Pathogens	IgG		IgM	
	Positive	Negative	Positive	Negative
*TOX*	722 (26.5%)	2004 (73.5%)	10 (0.4%)	2716 (99.6%)
*RUV*	2579 (94.6%)	147 (5.4%)	13 (0.5%)	2713 (99.5%)
*CMV*	2718 (99.7%)	8 (0.3%)	16 (0.6%)	2710 (99.4%)

## Data Availability

The data used to support the findings of this study are included in the article.
